# Lymphangioma circumscriptum of the scrotum following vasectomy

**DOI:** 10.4103/0970-1591.65408

**Published:** 2010

**Authors:** Dilip Kumar Pal, Manju Banerjee, Dhrubajyoti Moulik, Biplab Kumar Biswas, Manoj Kumar Choudhury

**Affiliations:** Department of Urology, Bankura Sammilani Medical College, Bankura, West Bengal, India; 1 Department of Surgery, Bankura Sammilani Medical College, Bankura, West Bengal, India; 2 Department of Pathology, Bankura Sammilani Medical College, Bankura, West Bengal, India

**Keywords:** Lymphangioma circumscriptum, scrotum, vasectomy

## Abstract

Lymphangioma circumscriptum is a congenital lymphatic hamartoma and rarely occurs in the male genital organs. Here we report a case of acquired lymphangioma circumscriptum of scrotum following vasectomy, which has not been reported till date. High clinical suspicion index is the clue to the clinician for diagnosis; histopathological confirmation and adequate surgical excision with deep lymphatic cisterns give the best result.

## CASE REPORT

A 45-year-old man was presented with complaints of fluid-filled lesions in the scrotum since last 15 years. Sometimes the lesions ruptured and milky discharge from the scrotum was noticed. Prior to admission to the hospital, due to acute cellulitis, he had sudden increase in the swelling with pain and watery discharge from the scrotum. The lesions appeared 15 years back as asymptomatic small vesicles within two weeks of vasectomy operation that were very slowly growing over the years. During this time he visited several doctors, treated with several courses of antibiotics and topical ointments without any relief. There was no history suggestive of filariasis.

**Figure 1:**
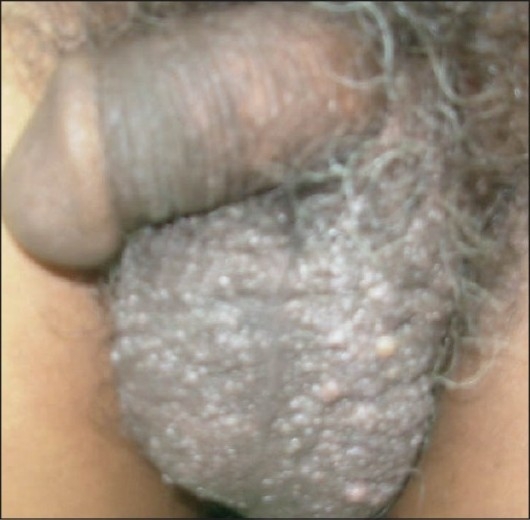
Whole scrotum except the neck is studded with multiple papules

On local examination, the whole of scrotum, except neck, was studded with multiple vesicles of variable size ranging from 1 to 8 mm [Figure 1]. On vesical aspiration some vesicles exuded clear fluid and some milky white fluid. There was no associated lymphadenopathy or lymphedema. Ultrasonography of the scrotum suggested complex septate cysts with internal echogenicity and without any communication with the scrotal contents.

**Figure 2:**
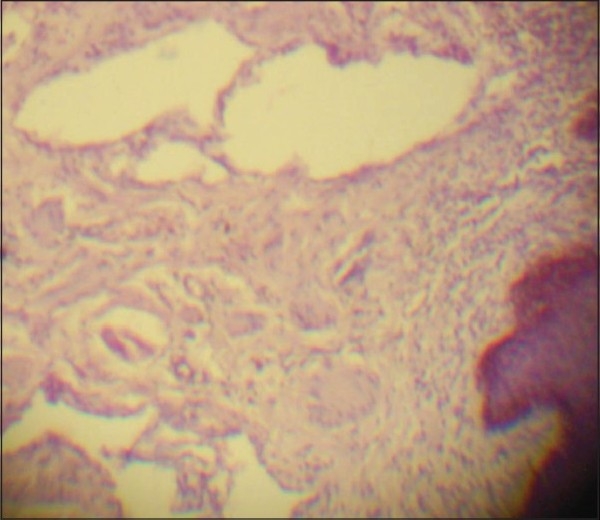
Histology showing multiple dilated subepidermal lymphatics (H and E, ×40)

The mass along with scrotal skin up to the tunica albuginea were excised together with ligation of all the lymphatic vessels. The skin was closed in two layers using the skin of the scrotal neck. The histological features were suggestive of multiple dilated lymphatic vessels lined by thin wall of endothelial cells with an inflammatory infiltrate in the papillary dermis [Figure 2]. Some of these dilated lymphatic vessels showed pinkish protenaceous material within it. The postoperative period was uneventful and on follow-up, there was no recurrence till one year.

## DISCUSSION

Lymphangiomas can occur anywhere in the skin and mucus membrane.[1,2] Although the common sites are axillary folds, shoulders, neck, proximal part of the limbs, tongue, and buccal mucus membrane,[1,2] the scrotum is the rarest site.[3–5] Even though the disease is congenital, rarely are there occurrence of acquired cases in the scrotum[3–5] and vulva.[6] The disease is characterized by translucent vesicles of varying sizes scattered or grouped like frog spawn, containing clear lymph fluid. These vesicles often are associated with verrucous changes, which give them the warty appearance. The basic pathologic process in the congenital cases, which are found at birth or early childhood are, collection of lymphatic cistern in the deep subcutaneous plane that are separated from the normal network of lymphatic vessels; however these lymphatic cisterns are connected with the superficial lymph vessels through vertical dilated lymph channels. Acquired lymphangioma circumscriptum develops later, probably due to injury or damage from the deep collecting channels in the tissue, leading to stasis of lymph with backflow resulting in subsequent dilation of upper dermal lymphatics causing the lesion.[6] These acquired cases are mostly due to infections like filariasis, lymphogranuloma venereum, tuberculosis, donovanosis, following trauma, surgery, or radiotherapy.[3,6] In the present case, there was a lymphatic obstruction, possibly due to ligation of draining lymphatics as a result of manhandling of tissue during vasectomy operation. Occurrence of the lesion within two weeks of vasectomy indicates lymphatic obstruction in the present case.

The disease is diagnosed on clinical index of suspicion and confirmed on histopathology. Ultrasonography and CT scan of the abdomen or pelvis are helpful in patients who have suspicious extensions of cystic lesion to the retroperitoneum or pelvis.[4] The pathologic findings are consistent with endothelial-lined canaliculi, connective tissue stroma filled with lymphocytes, monocytes, and polymorphonuclear cells.[4,6]

An adequate surgical excision up to feeding cisterns is the best option for treating such patients. The scrotal reconstruction can be achieved by free skin graft i.e., positioning a skin flap from the scrotum using skin of the scrotal neck alone or supplemented with a rotation flap from the skin of the thigh. The most common postoperative complication is its recurrence with an incidence of 25–50% within 3 months, which is usually due to improper surgical approach or inadequate excision of the tumor.[3–6] Other complications are edema, prolonged lymphatic drainage, and local infection. Although laser therapy with CO_2_ has been applied as an effective treatment[3,6] in such cases with good cosmetic result, therapeutic failure has already been reported.[6]

Even now adequate surgery is the only effective option for such patients in whom the disorder is persistent, disabling, and psychologically divesting.
